# Reply to R Kirwan et al.

**DOI:** 10.1093/cdn/nzac038

**Published:** 2022-03-16

**Authors:** David S Ludwig, Belinda S Lennerz, Jacob T Mey

**Affiliations:** New Balance Foundation Obesity Prevention Center, Boston Children's Hospital, Boston, MA, USA; Division of Endocrinology, Boston Children's Hospital, Boston, MA, USA; Integrated Physiology and Molecular Medicine, Pennington Biomedical Research Center, Baton Rouge, LA, USA

Dear Editor:

Kirwan et al. (1) highlight important limitations of our study, including selection bias, recall bias, and lack of generalizability. They advise caution in translating the results of our survey, which includes self-reported data, on the health effects of a carnivore diet. We agree with these concerns, as explicitly stated in our report (2). However, several specific criticisms of our study relate more broadly to scientific surveys and to nutrition research in general.

Regarding points of disagreement, we recognize that FFQs (and other dietary survey instruments) may correlate well with meat consumption in the general population consuming mixed diets. However, validation studies of these instruments would not have included a population similar to the one we studied, and the level of dietary detail available in standard FFQs pertaining to meat consumption patterns were insufficient for our purposes. For these reasons, we developed a survey instrument in consultation with members of the carnivore community, consistent with the principles of community-based participatory research.

Kirwan et al. mention the existence of “ideological echo chambers” that could “increase the likelihood of information gerrymandering” among our sample – a concern that could apply to many surveys of special communities, both within and outside the field of nutrition. However, we saw no evidence of co-ordination among respondents. Indeed, considerable variability exists among our data, including in health measures that might be considered derogatory to this dietary pattern. For example, many respondents reported markedly elevated LDL cholesterol – a commonly recognized cardiovascular disease risk factor – and a substantial increase on the carnivore diet compared with prediet levels. Along similar lines, Kirwan et al. speculate that the 28 duplicate entries (∼1% of the total), may indicate an attempt by some individuals to contravene study procedures and bias the data. A more benign explanation involves technical issues, such as uncertainty that a completed survey was successfully submitted online. In support of this possibility, none of the eliminated duplicate surveys had been fully completed by the respondents.

Kirwan et al. highlight potential misrepresentation of our research by media and lay outlets, but this issue is by no means specific to our study. As mentioned above, we extensively considered study limitations, including in the abstract, and noted potential harms affecting consumers (elevated LDL cholesterol, micronutrient deficiencies), animals, and the environment. Furthermore, we did not issue a press release or make any health claims about the carnivore diet on social media. Although researchers should take care not to exaggerate data in any public format, we do not think that discussion of scientific findings in an academic journal should be censored out of concern for possible misrepresentation by media or other lay outlets.

Regarding the larger context, scores of dietary surveys are published every year on various dietary patterns, many that utilize self-reported data to characterize health status. Should this study design be abandoned entirely? If so, how would one obtain initial descriptive information on special diets, including potential health/safety issues, and preliminary data to inform well-funded prospective studies and trials?

Despite the acknowledged limitations, our study has notable strengths in comparison to typical survey-based reports, including a large participant number and the focus on a dietary pattern about which little is known. In contrast to the several hundred surveys of vegan and vegetarian diets in the medical literature, few if any systematically collected data on the carnivore diet exist. Despite the self-report nature, data were consistent with expectations. Specifically, anthropometric and laboratory data were within physiologically plausible ranges and serum lipids showed the low triglycerides to HDL cholesterol ratio and high LDL cholesterol characteristic of diets with low carbohydrate and high saturated fat content (e.g. carnivore diet) – providing evidence pertaining to the validity of survey responses.

In conclusion, we reiterate the preliminary nature of our data and support the call by Kirwan et al. for high-quality research into the long-term positive and adverse health effects of a carnivore diet. We hope our study will motivate funding agencies in this regard.

## Notes

Author disclosures: DSL received royalties for books that recommend a carbohydrate-modified diet, and his spouse owns a nutrition education and consulting business. All other authors report no conflicts of interest.

Author contributions—all authors participated in drafting the letter and approved the final version. DSL and BSL had primary responsibility for the final content.
